# Synergizing community-based forest monitoring with remote sensing: a path to an effective REDD+ MRV system

**DOI:** 10.1186/s13021-017-0087-8

**Published:** 2017-12-01

**Authors:** M. S. R. Murthy, Hammad Gilani, Bhaskar Singh Karky, Eklabya Sharma, Marieke Sandker, Upama Ashish Koju, Shiva Khanal, Mohan Poudel

**Affiliations:** 1 International Centre for Integrated Mountain Development, GPO Box 3226, Kathmandu, Nepal; 2 Department of Atmospheric Sciences, University of Illinois, Urbana, IL 61801 USA; 3 UNREDD Program, United Nations Food and Agriculture Organization, Viale delle Terme di Caracalla, 00150 Rome, Italy; 4 Key Laboratory of Digital Earth Science, The Institute of Remote Sensing and Digital Earth (RADI), Chinese Academy of Sciences (CAS), Beijing, China; 5 Department of Forest Research and Survey, Babarmahal, Kathmandu Nepal; 6 REDD Implementation Center, Babarmahal, Kathmandu Nepal

**Keywords:** Satellite images, Community monitoring, REDD+ MRV, Biomass map, Nepal

## Abstract

**Background:**

The reliable monitoring, reporting and verification (MRV) of carbon emissions and removals from the forest sector is an important part of the efforts on reducing emissions from deforestation and forest degradation (REDD+). Forest-dependent local communities are engaged to contribute to MRV through community-based monitoring systems. The efficiency of such monitoring systems could be improved through the rational integration of the studies at permanent plots with the geospatial technologies. This article presents a case study of integrating community-based measurements at permanent plots at the foothills of central Nepal and biomass maps that were developed using GeoEye-1 and IKONS satellite images.

**Results:**

The use of very-high-resolution satellite-based tree cover parameters, including crown projected area (CPA), crown density and crown size classes improves salience, reliability and legitimacy of the community-based survey of 0.04% intensity at the lower cost than increasing intensity of the community-based survey to 0.14% level (2.5 USD/ha vs. 7.5 USD/ha).

**Conclusion:**

The proposed REDD+ MRV complementary system is the first of its kind and demonstrates the enhancement of information content, accuracy of reporting and reduction in cost. It also allows assessment of the efficacy of community-based forest management and extension to national scale.

**Electronic supplementary material:**

The online version of this article (10.1186/s13021-017-0087-8) contains supplementary material, which is available to authorized users.

## Background

The reliable monitoring, reporting and verification (MRV) of anthropogenic greenhouse gas emissions and removals from the forest sector is one of the most important elements necessary for the implementation of a performance-based reducing emissions from deforestation and forest degradation (REDD+) mechanism [1–3]. A few of the critical challenges of the REDD+ MRV systems are the reliable estimation of deforestation, afforestation, forest enhancement and degradation [3]. Currently, satellite-based monitoring systems are in vogue for the monitoring of forest cover gain and loss, facilitating the estimation of carbon fluxes from deforestation and afforestation [4].

On the other hand, using remote sensing to quantify carbon fluxes associated with forest enhancement and degradation in forest land remaining as forest over the given monitoring period is not possible on an operational scale, due to the complexities involved in detection and quantification [5]. In many developing countries, there is a low capacity for remote sensing-based monitoring and reporting of emissions from degradation (and removals from regrowth and afforestation) on a national level [6]. Estimates of above-ground biomass (AGB tons of dry weight) gain or loss in these cases are best collected by either forest inventory or production consumption surveys [7]. However, there is also a lack of government-endorsed programs that instill a dedicated effort for consistently monitoring forests on a national scale [7], due to the cost and time intensiveness of the field inventories.

In this context, Danielsen et al. [8] and Palmer [9] have explained the role of community-based forest monitoring systems and presented experiences from different countries in terms of optimizing field inventories, accuracy, economics, legitimacy, coupling and scalability to national systems. There is a very strong linkage between indigenous peoples and community forestry with less deforestation, degradation and improved enhancement [10–12]. The community-based monitoring systems involve scientifically identified, permanent sample plots over individual community forest (CF) areas, protocol development, training and periodic measurements of different parameters at specified intervals [13].

A few critical challenges of these community-based monitoring systems include the efficacy of high-intensity annual monitoring using permanent sample plots, lack of spatial extrapolation power to address changes beyond the sample plots, such as forest cover loss and degradation, and the reliable quantification of carbon dynamics over the entire study area [14]. Danielsen et al. [15] stressed the need for reliable third-party evaluations with a detailed explanation of the potential disadvantages of community monitoring, such as biased reporting by communities, intimidation of communities for biased reporting and over-burdening communities with workloads from state-owned systems [9]. Skutsch et al. [16] and Bavikatte and Jonas [17] stressed the need for the rational integration of geospatial technologies to improve the efficacy of community monitoring systems. Remote-sensing-based monitoring of forest degradation/enhancement essentially involves two main approaches [18]: first, detection indicated by a change in canopy cover or proxies and second, the quantification of gain or loss in AGB. Both approaches provide spatially explicit estimates with wall to wall coverage, enabling the understanding of dynamics beyond point-based estimates [4, 19]. The reliable remote-sensing-based visual indicators and systematic information systems could also extend objectivity to verification and third-party evaluations [1, 20]. The multi-resolution satellite systems help to address biomass estimations at the species, stand and forest type levels, enabling the spatial linking of different scales of information to reach from the community level to national estimates [21, 22].

Against this background, the present study is conducted with a focus to develop operationally feasible synergistic community forestry monitoring approach drawing the strengths and benefits of both remote sensing and community monitoring resulting in higher integrated value of salience, reliability and legitimacy as against standalone methods. We present very-high-resolution satellite-based tree cover parameters, including crown projected area (CPA), crown density and crown size classes, and discuss how these work as simple indicators for monitoring CFs, as well as their potential use by communities. This paper also demonstrates the development of remote-sensing-based temporal spatial biomass estimates with better accuracies using optimized community-based field inventories. The added advantage of spatially explicit biomass dynamics in understanding leakage, additions and persistence are demonstrated through the comparative evaluation of CF areas, where conservation practices are followed with non-CF areas. We also show, through comparative scoring, how the use of community monitoring in conjunction with remote sensing could create more value when added to the MRV system in terms of salience, reliability and legitimacy.

## Methods

### Study site description

**Fig. 1:**
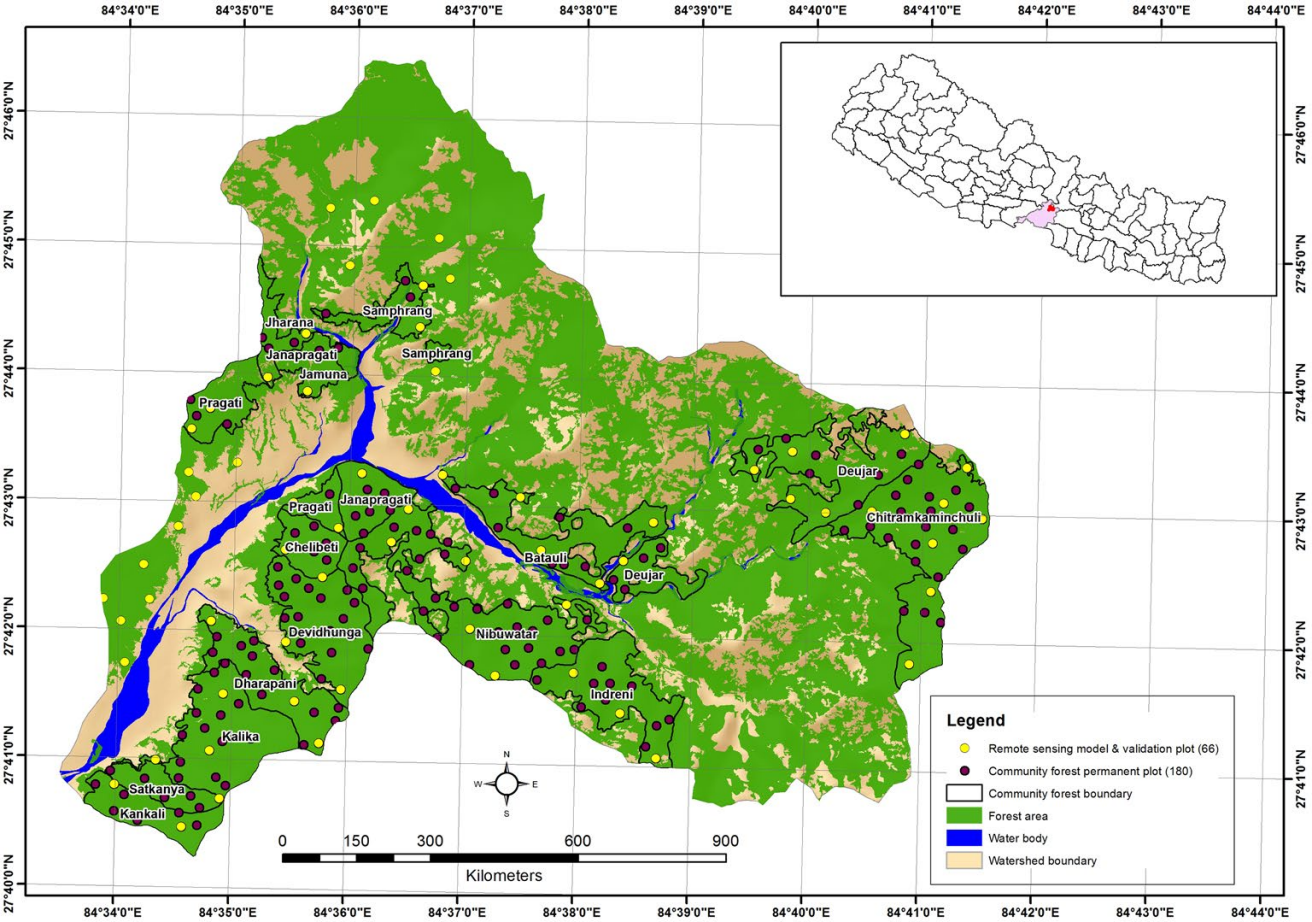
Study area map

We conducted the study in the Kayarkhola watershed of the Chitwan District, Nepal (Fig. 1). The total study area is 8002 ha, in which 2385 ha is under 16 community forestry (CF) units owned by community forest user groups (CFUG) and 5617 ha is under non-CFs (government and leasehold forests, including miscellaneous land cover). The study area is located at a latitude of 27.668–27.776 and a longitude of 84.556–84.695 [23]. The Kayarkhola watershed is one of three watersheds in Nepal where the REDD+ pilot project (2009–2013) has been implemented. Under the Forest Carbon Trust Fund (FCTF), 16 CFUGs received seed grants of US $21,905 in 2011 [24], US $24,691 in 2012 [25] and US $25,659 in 2013 [26].

### Datasets

To assess and monitor the forest parameters in this study, orthorectified IKONOS-2 (August 3, 2002) and GeoEye-1 (November 2, 2009, and December 15, 2012) satellite images were used at 1 and 0.5 m spatial resolution, respectively.

Watershed boundary delineation was completed through spatial analysis using a 20 m-resolution topographic digital elevation model (DEM). A GIS-based participatory approach was used for the boundary delineation of the 16 CFs involving CFUGs and a district forest officer (Fig. 1 and Additional file 1: Appendix S1). The CF area was subtracted from the watershed boundary, to extract biophysical parameters for both CF and non-CF regimes.

### Land cover assessment and change analysis

Tree cover area was extracted using the GEOgraphic object-based image analysis (GEOBIA) technique [27], performed on high-resolution satellite images (IKONOS and GeoEye-1) to delineate tree cover and non-tree cover areas. The segmentation parameters used include scale—400, shape—0.7, and compactness—0.3. The non-tree cover areas were further classified as agriculture, settlements, barren areas and water. The 2009 land cover map was used as a reference to classify the 2002 and 2012 satellite images. The tree cover loss and gain were estimated for the periods of 2002–2009 and 2009–2012 using the change matrix method [28].

### Tree cover assessment: forest change analysis

Tree cover assessment was completed using three parameters: (1) crown projected area (CPA), (2) crown density and (3) crown sizes. CPA represents the aggregate area of all tree crowns vertically projected onto the ground surface. The sum of crown areas for all trees was measured on a fixed plot area and then divided by the ground area of the plot to give the crown density or closure. All 1 ha grids covering more than 10% tree crown density were treated as forests, and the remainder were < 10% crown density. Later, all forested grids were further classified into three forest crown density classes of 10–40%, 40–70% and > 70%. The transformation to higher crown density classes was considered an enhancement, while transformation to lower crown density classes was considered a degradation (Methodology details in Additional file 1: Appendix S2). The forests of Nepal are reported to be of middle age with less complex canopies [29], as observed over the study area, with trees largely belonging to the less than < 100 cm diameter class (Additional file 1: Appendix S3). Accordingly, in terms of the progression in crown size classes during 2002–09 and 2009–12 across the watershed, CF and non-CF areas were analyzed for use as proxy indicators of forest enhancement.

### Field-based biomass estimations

The field-based biomass estimations were obtained from two sets of samples. The first set belongs to the community measurements, where the members of CFUGs carried out field measurements annually in permanent sample plots from 2009 to 2013. A total of 140 permanent circular sample plots (area 250 m^2^ and radius 8.92 m) covering 3.5 ha were established for field measurement in all CFs, with a sampling intensity of 0.147% (Fig. 1).

The approach for the second set of samples was focused on building the CPA-biomass model and to carry out the spatial biomass estimation for the entire watershed area. Based on the overall understanding gained from community-based field data on the variability of biomass, we have taken approximately 1/3 of the community field sampling intensity (51 out of 140) to cover 2.55 ha of forest area in the entire watershed with a sampling intensity of 0.043%. Additionally, we have increased the size of the plot to a 500 m^2^ circular plot to obtain more local variance. Field campaigns were conducted from December 2009 to December 2010.

In each plot for both sampled streams, tree parameters including DBH (only > 5 cm), height, crown diameter, and species were recorded in addition to geographic coordinates. A GPS receiver for location identification, TruPulse 360B for tree height measurement and diameter tape for DBH measurement were all used in the field. Plot-level AGB values (in tons) were calculated using the allometric equation given by Chave et al. [30] for tropical, moist hardwood forests, which were upscaled at 1 ha. Wood specific gravities for each tree species were taken from data published by Chaturvedi et al. [31].

### Spatial AGB model

For spatial AGB, a linear regression relationship was developed using field-based biomass data from the center of 1 ha grid and CPA obtained from the 2009 GeoEye-1 image. The model was calibrated and validated using 31 plots (60%) and 20 plots (40%), respectively, based on measured AGB values from the field data. A significant correlation coefficient allowed us to use the same linear regression model to estimate the biomass using CPA maps for the entire study area for 2002 and 2012. Based on individual time spatial AGB maps, biomass spatial change maps at the two time intervals of 2002–2009 and 2009–2012 were prepared to assess the gain and loss of biomass over the entire watershed, for both CF and non-CF areas.

### Comparison of remote-sensing-based and field-based AGB

The community-based permanent field plot measurements have given us information on AGB (tons/ha) and the mean annual increment over 140 locations for the years 2009 and 2012. From the biomass maps of 2009 and 2012 over these 140 locations, remote sensing model-based biomass estimates were extracted and then compared with community-based biomass values, with the root mean square error determined to analyze the statistical significance.

### REDD+ MSRL index

The aspects governing the operational feasibility and sustainability of our approach and proposed framework were analyzed using salience, reliability and legitimacy parameters as identified by Danielsen et al. [15]. Salience reflects how knowledge outcome answer right question, outcome provided is in useful form and time, offers an opportunity to relate results to policies and actions. The contextualization in terms of adopting local context, potential to couple with national systems, linkages to performance and support to undertake diagnostic/prescriptive actions are the variables taken to assess salience. The reliability refers to meeting standards of scientific plausibility, technical adequacy about data, methods used, analysis applied and robustness of conclusions. The information content, accuracy, cost effectiveness and repeatability are the variables taken to assess reliability. Legitimacy offers an opportunity for involvement of stakeholders with appropriate mechanisms to facilitate the expression of values and the resolution of conflicts with end users. The potential to remove bias, transparency of the process, scope for participatory process and mutual trust among the stakeholders involvement are the variables used for legitimacy assessment [8, 15, 32].

The different variables selected as above for salience, reliability and legitimacy in accordance with REDD+ monitoring context were ranked on 1–3 scale (Table 4). The score values were assigned based on the tools and methods used and their output characteristics as explained in Table 4 for each variable. In order to assess the integrated effect of all the parameters and assess potential adoptability, we developed the REDD+ MSRL Index (Monitoring with salience reliability legitimacy) using the following formula:

$${\text{REDD + MSRL index }}({\text{Monitoring with salience reliability legitimacy}}) = \frac{{\sum\nolimits_{n = 1}^{12} {( {\text{S}}_{\text{i}})} }}{{\sum\nolimits_{n = 1}^{12} {( {\text{Max}}_{\text{i}})} }},$$

where *S*_*i*_, is the score of the *i*th variables of salience, reliability and legitimacy, i = 1,2, …12 and *Max*_*i*_ = *Max {S*_*i*_ *}*

## Results

### Land cover change analysis

**Fig. 2:**
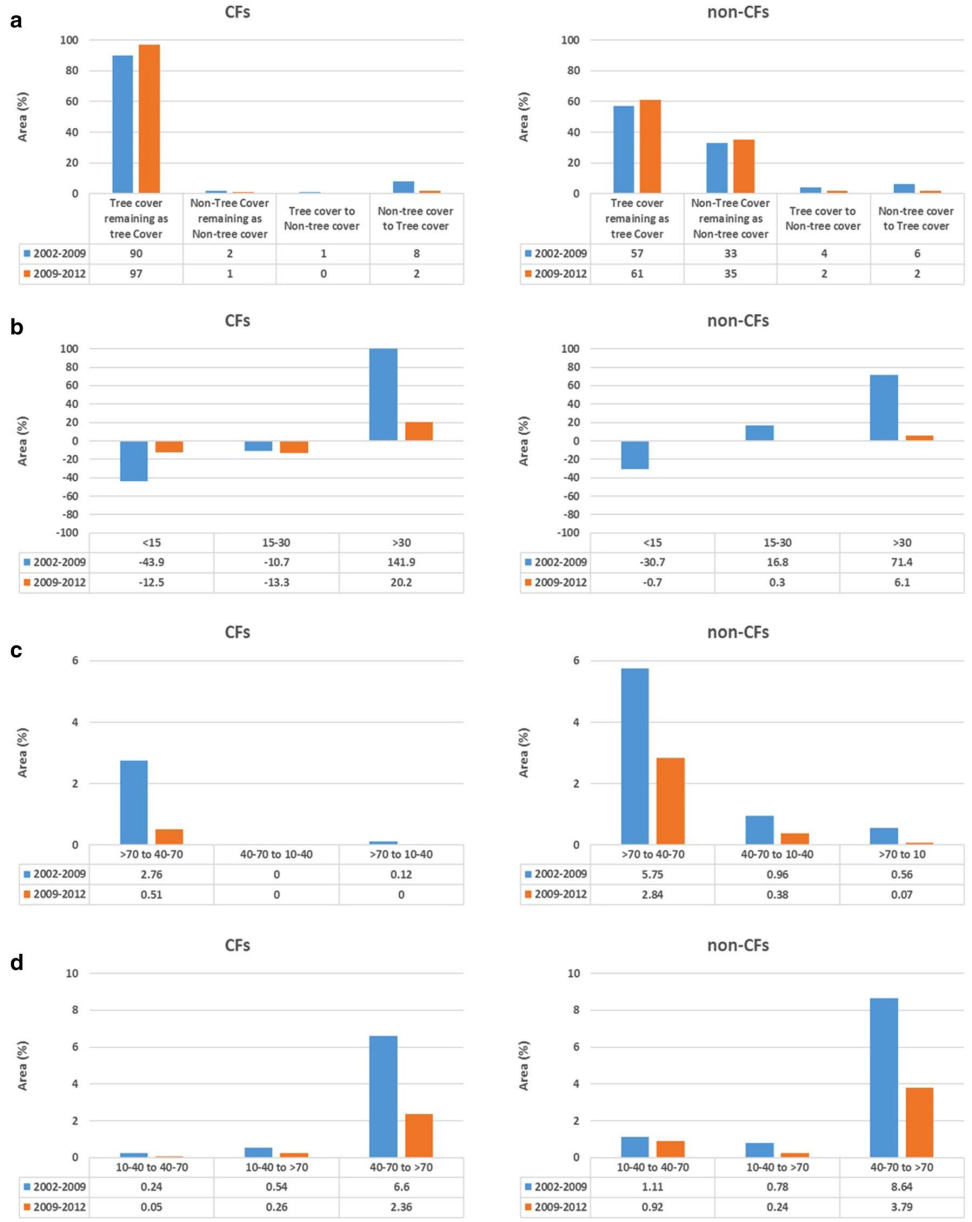
Changes in different biophysical parameters extracted from satellite data during 2002–2009 and 2009–2012, in CFs and non-CFs areas. **a** Land cover change. **b** Tree crowns size (m^2^) change. **c** Lower crown density class. **d** Higher crown density class

In the study area, the total tree cover area has increased from 5584 to 5864 ha during 2002–2009 and to 5892 ha by 2012 at the watershed level. A similar increase is also found in both CF and non-CF areas of the watershed (Table 1). However, from the tree cover change dynamics (Fig. 2 and Additional file 1: Appendix S3), it can be found that CFs are stable and improved, compared to non-CF areas and changes at the watershed level. In the CF areas, 90% of the tree cover remained the same without conversion during the periods of 2002–09 and 2009–12. In addition, the conversion of non-tree cover into tree cover has resulted in an 8% increase, and tree cover to non-tree cover only increased by 1% in CF areas.

**Table 1:** Status of different monitoring parameters extracted from satellite data 2002, 2009, 2012

	Area (ha)		
	2002	2009	2012
1. Land cover			
Watershed			
Tree cover with < 10% crown density	1221	991	975
Tree cover with > 10% crown density (Forest)	4363	4873	4917
*Subtotal: tree cover*	5584	5864	5892
Agriculture and built-up area	2110	2021	2021
Barren area	272	83	56
Water body	36	34	33
Total watershed	8002		
CFs			
Tree cover with < 10% crown density	500	376	378
Tree cover with > 10% crown density (Forest)	1667	1946	1976
*Subtotal: tree cover*	2167	2322	2354
Agriculture and built-up area	49	34	26
Barren area	167	26	3
Water body	2	3	2
Total CFs	2385		
Non-CFs			
Tree cover with < 10% crown density	721	615	598
Tree cover with > 10% crown density (forest)	2696	2927	2940
*Subtotal: tree cover*	3417	3542	3538
Agriculture and built-up area	2061	1987	1995
Barren area	105	57	53
Water body	34	31	31
Total Non-CFs	5617		
2. Tree crown size			
Watershed			
< 15 m^2^	2191	1453	1454
15–30 m^2^	2024	2228	2247
> 30 m^2^	1369	2183	2191
Total	5584	5864	5892
CFs			
< 15 m^2^	843	575	581
15–30 m^2^	816	887	901
> 30 m^2^	508	860	872
Total	2167	2322	2354
Non-CFs			
< 15 m^2^	1348	878	872
15–30 m^2^	1208	1341	1347
> 30 m^2^	861	1323	1319
Total	3417	3542	3538
3. Forest crown density (%)			
Watershed			
10–40	3631	3885	3919
40–70	512	717	726
> 70	220	271	272
Total	4363	4873	4917
CFs			
10–40	1391	1547	1571
40–70	193	286	291
> 70	83	113	115
Total	1667	1946	1976
Non-CFs			
10–40	2241	2338	2348
40–70	318	432	435
> 70	137	157	157
Total	2696	2927	2940
4. Above ground biomass (AGB)			
Watershed			
Total AGB (ton)	2,661,790	2,928,074	2,985,291
Average AGB (ton/ha)	342	354	360
Standard deviation AGB	121	123	130
CFs			
Total AGB (ton)	1,120,050	1,236,838	1,265,923
Average AGB (ton/ha)	370	392	405
Standard deviation AGB	103	103	110
Non-CFs			
Total AGB (ton)	1,541,740	1,691,236	1,719,368
Average AGB (ton/ha)	324	331	367
Standard deviation AGB	128	128	134

### Forest crown density change: enhancement and degradation

The areas under all forest crown density classes were found to have increased during the periods of 2002–09 and 2009–12 in both the CF and non-CF areas, signifying a net positive growth of forests in the watershed (Table 1). The enhancement is largely due to the conversion of the 40–70% class into the > 70% crown density class, in both CF and non-CF areas. The degradation is found to be highest in the non-CF areas, where 155 ha of the > 70% crown density class is converted into the 40–70% crown density class (Fig. 2 and Additional file 1: Appendix S3).

### Assessment of CPA and tree crown sizes

An accuracy of 83% was achieved in the delineation of tree crowns, as observed through the relationship determined between the number of segmented and observed tree crowns (Additional file 1: Appendix S5). Spatial maps over the watershed can be seen in Additional file 1: Appendix S6. The CPA in the higher crown size class of > 30 m^2^ was found to have increased by 59.4% during 2002–2009 and by 0.4% during 2009–2012 at the watershed level, with a total area increase of 872 ha over the > 30 m^2^ crown size class, indicating a positive growth of forests (Table 1).

### Comparison of remote-sensing-based and field-based AGB

**Fig. 3:**
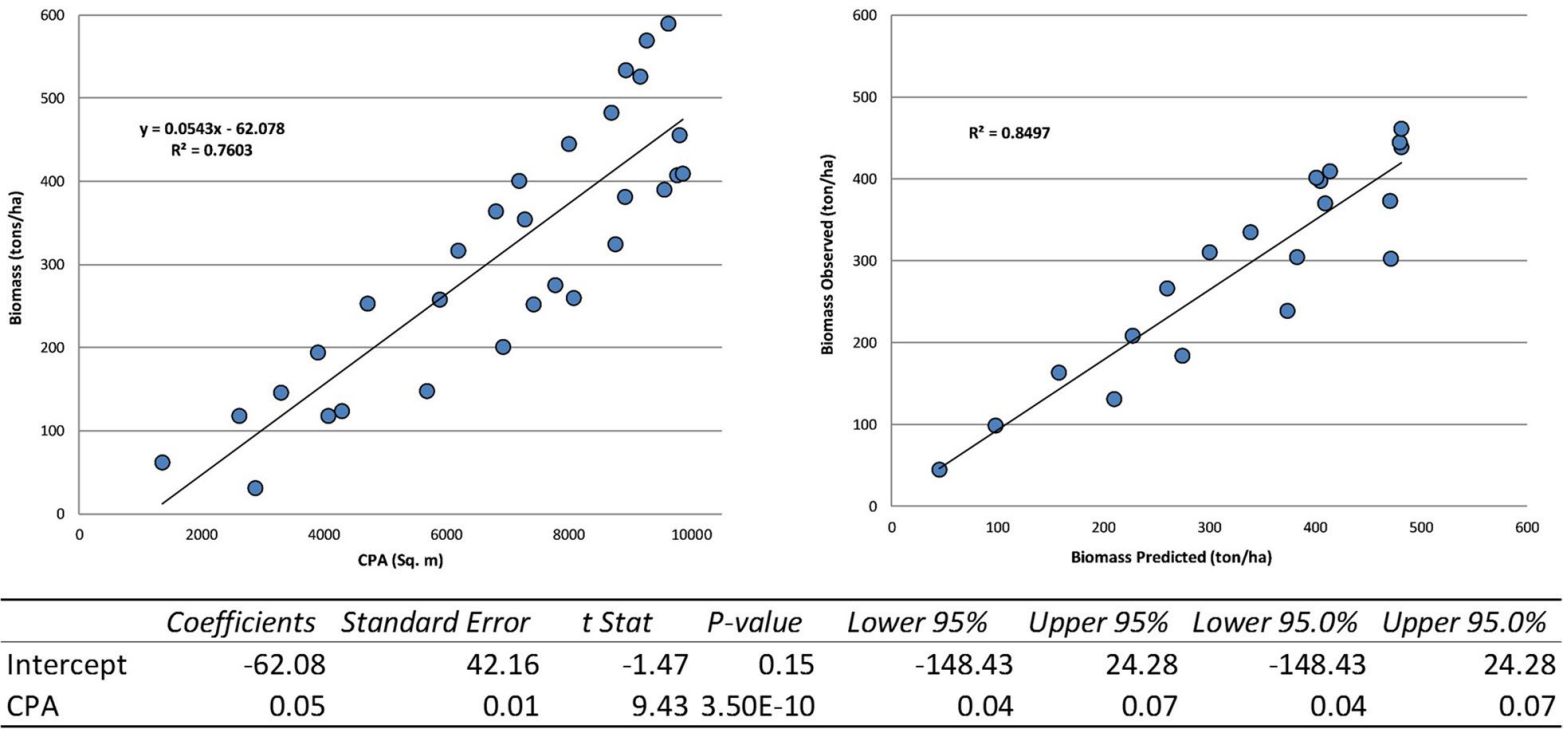
(Left side scatterplot) A linear regression model fitting between CPA and biomass values, (right side scatterplot) validation of biomass model and (bottom table) the statistical values of linear regression model

A linear regression model was obtained to map AGB at the watershed level (AGB = 0.0543 * CPA − 62.078 with R^2^ = 0.76). An 84% accuracy was achieved between the predicted and observed biomass values using 20 sample locations (Fig. 3). AGB values from maps (Additional file 1: Appendix S7) were extracted over 140 sample plot locations where community inventory-based biomass estimations were available. The estimates were aggregated over 15 CFs (One CF was excluded from the analysis because that was not delineated in 2009 and thus not permanently observed by CFUG) using the corresponding sample plots for each CF (Table 2). The annual increase in biomass of 2.37 tons/ha was estimated by the community-based inventory, whereas the CPA model-based estimates were 2.61 tons/ha with a 0.95 tons/ha root mean square error (Table 2).

**Table 2:** Comparison of field-based and remote-sensing based AGB values in the community forests in Kayar Khola watershed in 2009 and 2012 and change over time

No	CF name	No. of field sample plots used	Field based AGB						RS based AGB					
			Total 2009 (ton)	Av. 2009 (ton/ha)	Total 2012 (ton)	Av. 2012 (ton/ha)	Annual change (ton)	Annual change/ton/ha	Total 2009 (ton)	Av. 2009 (ton/ha)	Total 2012 (ton)	Av. 2012 (ton/ha)	Annual change (ton)	Annual change/ton/ha
1	Batauli	11	3873.15	352.10	3895.23	354.11	7.36	0.67	3883.26	353.02	3917.06	356.10	11.27	1.02
2	Chitramkaminchuli	8	1639.53	204.94	1852.50	231.56	70.99	8.87	1664.86	208.11	1873.62	234.20	69.58	8.70
3	Deujar	9	3016.15	335.13	3075.33	341.70	19.73	2.19	3051.04	339.00	3104.26	344.92	17.74	1.97
4	Devidhunga	29	12,931.54	445.92	12,990.13	447.94	19.53	0.65	12,982.21	447.66	13,235.16	456.38	84.32	2.91
5	Dharapani	13	5361.57	412.43	5421.76	417.06	20.06	1.54	5428.09	417.55	5488.97	422.23	20.29	1.56
6	Indreni	6	2110.68	351.78	2187.88	364.65	25.73	4.29	2143.34	357.22	2205.79	367.63	20.82	3.47
7	Jamuna	3	1108.11	369.37	1118.03	372.68	3.31	1.10	1111.42	370.47	1133.41	377.80	7.33	2.44
8	Janapragati	7	1747.81	249.69	1819.56	259.94	23.92	3.42	1765.87	252.27	1852.81	264.69	28.98	4.14
9	Jharana	5	2013.51	402.70	2031.35	406.27	5.95	1.19	2020.88	404.18	2038.34	407.67	5.82	1.16
10	Kalika	7	2355.69	336.53	2383.48	340.50	9.26	1.32	2392.01	341.72	2434.02	347.72	14.00	2.00
11	Kankali	4	927.68	231.92	947.41	236.85	6.58	1.64	975.08	243.77	983.36	245.84	2.76	0.69
12	Nibuwatar	21	8900.92	423.85	9083.08	432.53	60.72	2.89	8942.85	425.85	9080.94	432.43	46.03	2.19
13	Pragati	7	1899.60	271.37	1951.34	278.76	17.25	2.46	1969.84	281.41	2004.77	286.40	11.64	1.66
14	Samfrang	6	2454.06	409.01	2463.94	410.66	3.29	0.55	2443.32	407.22	2481.75	413.63	12.81	2.14
15	Satkanya	4	1079.22	269.80	1112.74	278.18	11.17	2.79	1102.71	275.68	1139.99	285.00	12.43	3.11
Total		140	51,419.22	337.77	52,333.75	344.89	20.32	2.37	51,876.78	341.67	52,974.26	349.51	24.39	2.61

RMS error between field and RS based AGB Annual change/ton/ha = 0.95

### Spatial biomass change

The AGB during 2002–2009 and 2009–2012 increased by 116,788 and 29,085 tons, respectively, over CF areas with an annual increase of 7.7 and 4 tons/ha during 2002–09 and 2009–2012, respectively (Additional file 1: Appendix S4). The assessment over the watershed area, including CF and non-CF areas, reveals that the study area has largely acted as a sink through the increased biomass by 266,284 and 57,217 tons during 2002–09 and 2009–12, respectively.

### Cost comparison

The cost comparison of three different approaches (1) Community based monitoring (CM) without using remote sensing (2) Remote Sensing + Professionals based monitoring (RS + P) and (3): Remote Sensing + Community based monitoring (RS + CM) is presented in Table 4. The cost estimation involved three components satellite data cost, field inventory and image interpretation analysis. The community based monitoring for the year 2009 and 2012 costs USD16,000 covering CF area of 2385 ha with sampling intensity of 0.147%/year. Accordingly, the average per ha cost is found to be USD 7.5 ha. For the remote sensing based monitoring, optimal sampling intensity of 0.043% has been achieved using remote sensing based stratification across CF and non-CF areas covering the entire watershed for the year 2009. The CPA based biomass model developed for 2009 using this field data was used to generate biomass estimates for the year 2012 without further fieldwork. Hence the RS + P approach resulted in an average monitoring cost of USD 4/ha due to reduction in field sampling intensity for the year 2009 and avoiding sampling for the year the 2012 by using CPA based biomass model. The RS + CM based cost is found further reduced to USD 2.5/ha when the field inventory cost of professionals is replaced with the cheaper per plot cost of community based monitoring (Table 3). This reduced cost is realized despite including cost of satellite data for both the years and professional’s cost for data interpretation. Apart from realizing the cost benefits, the increased number of products and information generated using remote sensing approach is also presented in Table 3.

**Table 3:** Cost (USD) of forest monitoring (2009–2012) using different approaches over our study area—a comparative assessment using different approaches

Items	CM	RS + Professionals	RS + CM	Comments
Satellite data cost	Freely available Landsat 30 m data for stratification	3000 New tasking—1st time (2009)	3000 New tasking—1st time (2009)	
		1500 Archive Data (2012)	1500 Archive Data (2012)	
Field inventory cost	16,000 (inventory in 2009 and 2012 by communities) (Total number of plots 140 @ USD 115 per plot)	14,000 (inventory in 2012 by Professionals) (Total number of plots 51 @ USD 275 per plot)	5100 (inventory in 2012 by Communities) (Total number of plots 51 @ USD 100 per plot)	Based on 2012 reduced field sampling and remote sensing image, biomass model was developed to produce spatial biomass estimates for 2012. Based on this model and using satellite data of 2009, spatial biomass estimates for 2009 were made
				This has facilitated to avoid field sampling in 2009 and has reduced cost
Analysis (local professional)	2000	5000	5000	
Total cost (US $)	18,000	23,500	14,600	
Total area coverage (ha)	2385 (limited to CFUG area)	8002 (covers entire watershed)	8002 (covers entire watershed)	
Field sampling intensity (%)/year	0.147	0.043	0.043	
Cost (US $/ha)	7.5	4.0	2.5	Note that cost reduction/ha has is achieved due to increased study area and tenfold reduction in field sample intensity
Products	Field based biomass/ha for each CFUG for 2009 and 2012	Spatial products and area information on deforestation, afforestation	Spatial products and area information on deforestation, afforestation	
	Regeneration, disturbance information	Forest enhancement and degradation information in terms of changes in crown density classes, tree crown size class wise CPA	Forest enhancement and degradation information in terms of changes in crown density classes, tree crown size class wise CPA	
	No information on outside CF area	Spatial biomass maps facilitating change in biomass within and outside CF areas	Spatial biomass maps facilitating change in biomass within and outside CF areas	
	Non information on deforestation, afforestation beyond plot location			
	Degradation in terms of biomass loss is made from plot data over entire CFUG area			

*CM* community based monitoring, *RS* *+* *P* remote sensing +professionals based monitoring, *RS* *+* *CM* remote sensing +community based monitoring

**Table 4:** Comparative assessment (on 1–3 score value) of potential adaptability of Community based monitoring (CM) and Remote Sensing + Community based Monitoring (RS + CM) methods

No	Parameters	CM method ranking	RS + CM method ranking	Remarks
A. Salience				
1	Contextualization	High (3)	High (3)	Both the methods relies on local context of forest characteristics, measurements and change.
2	Coupling to national systems	Medium (2)	High (3)	RS + CM methods facilitate the concept of Danielsen et al. [15] on integration of local community monitoring through multi-scale approach
3	Linkages to performance	Medium (2)	High (3)	Due to spatial explicit wall–wall information, linking to payments becomes more reliable using RS + CM, and also addressing leakage
4	Diagnostic/prescriptive support	Low (1)	Medium (2)	RS + CM due to spatial character and synergy with local ground data helps planning for local prescriptions for forest management
B. Credibility				
5	Informative	Medium (2)	High (3)	RS + CM produces 70% of CM inputs with spatial explicitness to identify areas of positive, negative change, leakage over large area, CM limits to plot or limited traverses
6	Accuracy	High (3)	High (3)	Both produces > 80% accurate information
7	Cost effectiveness	Medium (2)	High (3)	RS + CM is estimated as less costly (Ref Table-4)
8	Repeatability	Medium (2)	Medium (2)	Risk of communities with drawing from measurements exists. RS + CM models need to be developed on region specific context, current approach given do not work for old growth forests
C. Legitimacy				
9	Removal of bias	Low (1)	Medium (2)	Intrinsic and extrinsic factors of CM potentially can induce bias [15]. RS + CM introduces bias due to interpretation/model inaccuracies but can be improved
10	Transparency	Medium (2)	High (3)	Geospatial methods known as best visualization tools, open access data and platforms, hence RS + CM is more transparent
11	Participatory	High (3)	Medium (2)	RS + CM builds models on community data, hence relatively extrinsic and might suffers from non participation
12	Mutual trust	High (3)	Medium (2)	RS + CM involves professionals and community, hence potential risks exists for mistrust, can taper down over time
REDD+ MSRL index:potential adoptability		0.72	0.86	

High = 3, Medium = 2 and Low = 1

### REDD + MSRL Index

The comparative score values against different variables of salience, reliability and legitimacy for Community based monitoring (CM) and Remote Sensing + Community based Monitoring (RS + CM) methods are presented in Table 4. Both the methods were scored with equal and high score values against contextualization. The variable for diagnostic/prescriptive support has got lowest score for CM method among all variables of salience. The RS + CM method is weighted with score value of 11 for salience against score value 8 of CM method. The variables on information content and cost effectiveness of reliability parameter are found with low score of CM method as compared to RS + CM method. The CM method is weighted with high scores for variables on participatory process and mutual trust. The integrated REDD+ MSRL Index of CM and RS + CM methods are 0.72 and 0.86 respectively.

## Discussion

Several studies have been conducted over our REDD+ pilot study areas in Nepal to assess the potential application of remote sensing to estimate tree canopy diameters, and these studies reported a strong relationship between CPA and ground measured biomass [33, 34]. The present study adds a temporal change component to these earlier studies through the conjunctive use of remote sensing and community inventories to develop salient, reliable and legitimate monitoring methodology.

### Remote sensing-based canopy indicators and biomass estimation: integration with community monitoring

This potential for canopy parameters to work as proxy indicators of the reduction/increase of biomass [18] reflects forest degradation/enhancement (Additional file 1: Appendix S6). Additionally, spatially explicit temporal CPA maps can provide strong secondary support for plot level community measurements, as a means for third-party evaluations or verification of changes within and outside CF areas.

As a proof-of-principle, we generated a range of CPA templates representing the basal area and analyzed the capacity of communities to understand such image templates (Additional file 1: Appendix S8) through a two-day field orientation workshop with 30 representatives of CFUGs. Here, we propose the use of Google Earth high-resolution temporal images and cost-effective software such as Easy Acreage (http://easyacreage.esoftfinder.com/) to develop simple operational packages for developing CPA-based maps and quantitative assessments.

The development of temporal biomass maps using the strong relationship between remote sensing-based CPA and community-based biomass measurements yielded two important useful outcomes in terms of extending the area under the assessment beyond community forest areas (Table 3), then adopting optimized field sampling only to develop a CPA-based biomass model and produce a model-based biomass maps for three periods. This has essentially eliminated community-based monitoring for 2002 and 2012 but to use CPA-based biomass model of 2009 to produce biomass maps for 2002 and 2012 and hence has optimized the cost (Table 3). Hence, the study clearly documents trade-offs that exist between monitoring costs and precision, and this translates to REDD+ benefits as a need for promoting effective REDD+ monitoring systems [15, 18].

### CF and non-CF change dynamics: an upscaling framework

We have up scaled our plot-level estimates to a watershed using CPA-based biomass model representing *Shorea robusta* and a mixed, broad-leaved forest type category, which could be further used to upscale across identical forest type under a similar bioclimatic region (Additional file 1: Appendix S9). The forest type map of Nepal DFRS [29] clearly depicts six major forest types, which are embodiments of climatic, physiographic and physiognomic expressions that provides a reliable framework for developing underneath stand level models and up scaling, as demonstrated in the study.

This kind of community driven remote sensing-based, bottom up sub-national assessment aggregated over the country could be explored for seamless flow into national monitoring systems to support national level forest reference level (FRL) for REDD+ and to build salience, reliablity and legitimacy for community-based inventory systems [8, 35]. For example, such an upward flow of community data into Landsat TM-based global forest monitoring systems [36] could contribute to the validation, improvement, and evolution of national forest monitoring systems.

### Operational feasibility analysis REDD+ MSRL index

The score values of different variables of salience, reliability and legitimacy have brought out the strengths and weaknesses of both the methods. The spatial explicit, synoptic, multi-scale nature and visualization benefits of remote sensing have added advantage for higher scoring of several variables for RS + CM method. The higher scores are gained especially for variables like coupling local systems to national systems, enhancing information content and cost-effectiveness. Our assessment indicates the need for improving the participatory process and mutual trust even after integrating community sampling into the remote sensing based approach to enhance the legitimacy of the system. This is due to the fact that RS + CM method involve professionals and communities of different disciplines and hence the potential risk for mistrust might arise on technicalities and capabilities leading to failure of effective implementation of the monitoring as a joint approach. Similarly, parameters like repeatability and removal of bias are found to be challenging variables for both the approaches owing to their inherent limitations as mentioned in Table 4. Our proposed REDD+ MSRL Index has essentially taken into account these variable strengths and weaknesses of different variables of salience, reliability and legitimacy reflecting higher index value of 0.86 of RS + CM method against 0.72 of CM method. Based on this, it can be concluded that RS + CM method enhanced the effectiveness of REDD+ monitoring. Even our community-based monitoring cost is also found to be along similar lines of experience elsewhere in the world (Additional file 1: Appendix S10).

It is to be noted that as part of developing REDD+ MRV system, the earlier studies were focused to develop ecologically sound forest change reporting databases and systems [23, 28, 37–41], understand and implement how communities perceive and practice scientific data and tools [8, 32, 42–45] and the challenges in evolving further technological improvements and capacity building towards institutionalizing such systems [46, 47]. However, our study has drawn strengths from understanding of individual streams of biophysical and social studies and developed unique method of integrating frontier technology based information with local community measurements and adopted novel way of testing its complementarity, efficacy and scalability using REDD+ MSRL Index. The proposed REDD+ MRV complimentary system is found enhancing information content and accuracy of change reporting, many fold reduction in cost, assessing efficacy of community forest regimes against non-community forest regimes, extending spatial framework to couple local systems to national monitoring systems. In this view, the study adds novelty to take forward in evolving socially acceptable REDD+ MRV systems and could draw necessary attention from policy makers for an appropriate uptake.

The recent developments in the global forest watch, upcoming regional cooperation systems such as SAARC for providing low-cost high-resolution satellite data, consortium-based approaches, open source software platforms, satellite data, innovative participatory and capacity-building tools can all extend an enormous scope to implant these systems among CFUGs to monitor and report forest changes [46]. Considering the great degree of promise reported in the success stories of the capacities of communities to produce accurate forest inventories [8], developing synergies with such emerging open source geospatial technologies could be a more positive endeavor to achieve in the near future.

## Conclusions

With the judicious and conjunctive use of approaches, remote sensing technology would be an additional, complementary asset for community-based forest monitoring, as well as for enhancing the effectiveness of REDD+ MRV systems. The high-resolution satellite-based tree cover monitoring products would be useful and strengthen third-party level evaluations. The national frameworks facilitating forward and backward coupling of the inventory systems could be leveraged from our proposed conjunctive approach to remote sensing and community monitoring systems. The global and regional cooperation and extended availability of open access geospatial services is strongly needed for testing and establishing such systems across different forest landscapes, as well as evolving as an approach with wider applicability.

## Additional file

**Additional file 1. Appendix S1:** Delineated community forest in Kayar Khola watershed, Chitawan based on handing over time.
