# Supplementary material for: Synergizing community-based forest monitoring with remote sensing: a path to an effective REDD+ MRV system
Source: Carbon Balance Manag. 2017 Dec 1;12:19. doi: 10.1186/s13021-017-0087-8 (PMC5711765; doi:10.1186/s13021-017-0087-8)
Supplement: Supplementary file 1 — Additional file 1. Appendix S1: Delineated community forest in Kayar Khola watershed, Chitawan based on handing over time. [file 13021_2017_87_MOESM1_ESM.docx]

**Appendix S1: Delineated community forest in Kayar Khola watershed, Chitawan based on handing over time.**

| **No** | **CF name** | **Digitized area (ha)** | **Handed over data** |
| --- | --- | --- | --- |
| 1 | Batauli | 156 | 31/10/2008 |
| 2 | Chitramkaminchuli | 314 | 22/03/2009 |
| 3 | Deujar | 279 | 23/12/2002 |
| 4 | Devidhunga | 192 | 27/10/2010 |
| 5 | Chelibeti | 65 | 27/10/2010 |
| 6 | Dharapani | 147 | 18/04/2007 |
| 7 | Indreni | 172 | 22/03/2009 |
| 8 | Jamuna | 35 | 21/09/2004 |
| 9 | Janapragati | 119 | 16/07/2003 |
| 10 | Jharana | 35 | Not available |
| 11 | Kalika | 214 | 15/07/2001 |
| 12 | Kankali | 92 | 19/09/1995 |
| 13 | Nibuwatar | 329 | 29/05/2006 |
| 14 | Pragati | 115 | 21/09/2004 |
| 15 | Samfrang | 64 | 23/09/2010 |
| 16 | Satkanya | 57 | 12/09/2004 |
| **Total** | | **2385** |  |

**Appendix S2: Methodology Details - Tree crown size and Crown Projected Area (CPA) estimation and mapping**

With effective conservation and protection measures, the forests are expected to result with improved stocks basically either resulting from increased number of trees (tree crowns) or enhanced tree growth (increased crown size). These two factors essentially could lead to increased Crown Projected Area (CPA). Accordingly, the study attempted detection and delineation of tree crowns, and estimation of total Crown Projection Area (CPA) was done using very high resolution satellite remote sensing data. Here we refer to ‘Tree Crown’ both for crown of an individual tree or overlapping crowns of more than one tree forming bigger coalesced tree crown. Our principle is to delineate such tree crowns using very high resolution satellite data and classify them into different size classes with a hypothesis that higher crown size class represent higher CPA at canopy level and beneath a larger tree or group of trees leading to higher basal area and hence higher biomass. We also put forth a proven scientific premise that forest moving into higher tree crown size classes represent positive growth of forests, improved age class and higher biomass. Since we are using one-meter resolution satellite data, it enables to analyze crown variability coming from individual tree crowns, coalesced overlapping tree crowns, shadows and canopy gaps using standard object based image classification techniques.

The GEOBIA Object based image classification system with a region growing technique was used for CPA detection and delineation at the watershed level. The scale-10, shape-0.7, and compactness-0.3 were used in segmenting IKONOS-2 and GeoEye-1 images. The image segmentation parameters were chosen using estimations from the scale parameter (ESP) tool proposed by [Drǎguţ et al. (2010)](#_ENREF_14). In the region growing technique method, tree tops are identified as maxima and the shadows between trees as minima. The segments are ‘grown’ from these maxima and the valleys act as boundaries. The first step in region growing was to create minimum size homogeneous objects through ‘chessboard segmentation’, the brightest pixels were then identified as seed pixels (tree tops). Regions were ‘grown’ from the seed pixels up to the local minima, resulting in homogeneous objects based on predefined homogeneity criteria ([Cui et al. 2008](#_ENREF_9); [Erikson 2003](#_ENREF_15); [Uddin et al. 2015](#_ENREF_38)).

The accuracy assessment of the delineated tree crowns was adopted from [Ke and Quackenbush (2011)](#_ENREF_25), i.e., a line-intercept transect method that plots the GEOBIA-determined CPA of trees with manually derived or ground measured CPA for quantifying the significant correlation coefficient. The accuracy of the CPA delineated through GEOBIA was assessed by comparing with the CPA derived from the images manually. Sixteen windows or grids (100 x 100 m) were randomly selected and tree crowns manually delineated and counted through image interpretation. The tree automatically delineated in the same grids using the region growing technique were also recorded. A linear regression line was plotted between the automatically delineated and manually counted numbers and the coefficient of determination calculated. We have chosen individual trees of different crown sizes with CPA estimated from ground and GEOBIA to assess the efficacy of region growing technique to identify the tree tops and boarders facilitating to draw crown perimeter.

**Appendix S3: Quantification of tree distribution of field based DBH ranges**


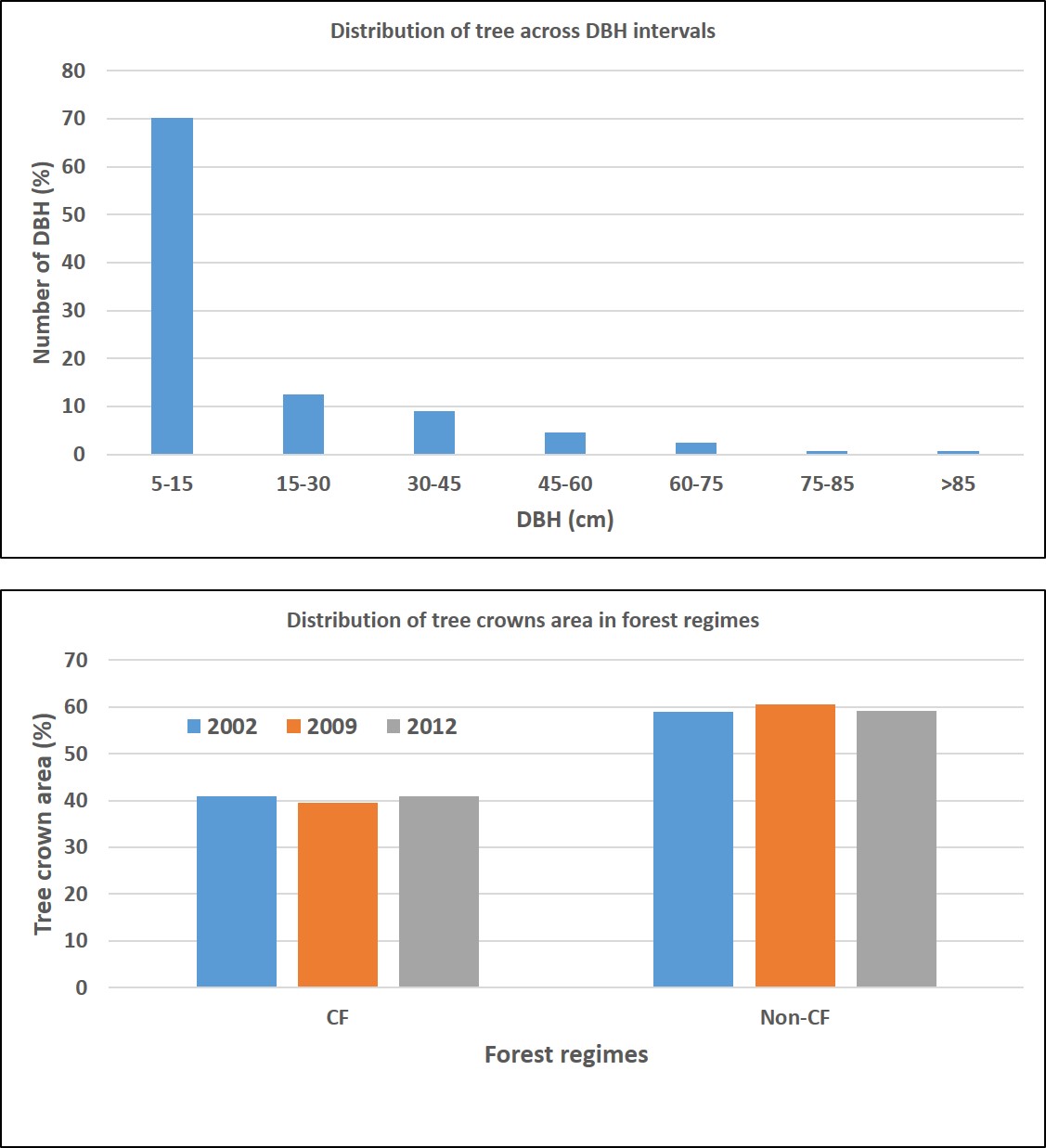


**Appendix S4: Change dynamics in different parameters extracted from satellite data during 2002-2009 and 2009-2012**

|  | | **Area (ha)** | | **Area (%)** | |
| --- | --- | --- | --- | --- | --- |
|  |  | **2002-2009** | **2009-2012** | **2002-2009** | **2009-2012** |
| **1. Tree cover change** | | | | | |
| **Watershed (8,002 ha)** | Tree cover remaining as tree Cover | 5,333 | 5,724 | 67 | 72 |
|  | Non-Tree Cover remaining as Non-tree cover | 1,887 | 1,970 | 24 | 25 |
|  | Tree cover to Non-tree cover | 251 | 140 | 3 | 2 |
|  | Non-tree cover to Tree cover | 531 | 168 | 7 | 2 |
| **CFs (2,385 ha)** | Tree cover remaining as tree Cover | 2142 | 2321 | 90 | 97 |
|  | Non-Tree Cover remaining as Non-tree cover | 38 | 30 | 2 | 1 |
|  | Tree cover to Non-tree cover | 25 | 1 | 1 | 0 |
|  | Non-tree cover to Tree cover | 180 | 33 | 8 | 2 |
| **Non-CFs (5,617 ha)** | Tree cover remaining as tree Cover | 3,191 | 3,403 | 57 | 61 |
|  | Non-Tree Cover remaining as Non-tree cover | 1,849 | 1,940 | 33 | 35 |
|  | Tree cover to Non-tree cover | 226 | 139 | 4 | 2 |
|  | Non-tree cover to Tree cover | 351 | 135 | 6 | 2 |
| **2. Area change under tree crows size class** | | | | | |
| **Watershed** | <15 m^2^ | -737.4 | -64.2 | -36.1 | -4.9 |
|  | 15-30 m^2^ | 102.7 | -90.3 | 5.4 | -4.5 |
|  | >30 m^2^ | 1088 | 283.2 | 99.9 | 13 |
| **CFs** | <15 m^2^ | -364.3 | -58 | -43.9 | -12.5 |
|  | 15-30 m^2^ | -84.7 | -94.1 | -10.7 | -13.3 |
|  | >30 m^2^ | 624.6 | 215.1 | 141.9 | 20.2 |
| **Non-CFs** | <15 m^2^ | -373 | -6.2 | -30.7 | -0.7 |
|  | 15-30 m^2^ | 187.4 | 3.8 | 16.8 | 0.3 |
|  | >30 m^2^ | 463.4 | 68 | 71.4 | 6.1 |
| **3. Forest enhancement (Shifts towards higher crown densities)** | | | | | |
| **Watershed** | 10-40 to 40-70 | 34 | 28 | 0.78 | 0.57 |
|  | 10-40 to >70 | 30 | 12 | 0.69 | 0.25 |
|  | 40-70 to >70 | 343 | 157 | 7.86 | 3.22 |
| **CFs** | 10-40 to 40-70 | 4 | 1 | 0.24 | 0.05 |
|  | 10-40 to >70 | 9 | 5 | 0.54 | 0.26 |
|  | 40-70 to >70 | 110 | 46 | 6.60 | 2.36 |
| **Non-CFs** | 10-40 to 40-70 | 30 | 27 | 1.11 | 0.92 |
|  | 10-40 to >70 | 21 | 7 | 0.78 | 0.24 |
|  | 40-70 to >70 | 233 | 111 | 8.64 | 3.79 |
| **4. Forest degradation (Shift towards lower crown densities)** | | | | | |
| **Watershed** | >70 to 40-70 | 201 | 93 | 4.61 | 1.91 |
|  | 40-70 to 10-40 | 26 | 11 | 0.60 | 0.23 |
|  | >70 to 10 | 17 | 2 | 0.39 | 0.04 |
| **CFs** | >70 to 40-70 | 46 | 10 | 2.76 | 0.51 |
|  | 40-70 to 10-40 | 0 | 0 | - | - |
|  | >70 to 10 | 2 | 0 | 0.12 | - |
| **Non-CFs** | >70 to 40-70 | 155 | 83 | 5.75 | 2.84 |
|  | 40-70 to 10-40 | 26 | 11 | 0.96 | 0.38 |
|  | >70 to 10 | 15 | 2 | 0.56 | 0.07 |
| **5. AGB change** | | | | | |
| **Watershed** | Total AGB change (ton) | 266,284 | 57,217 | 10 | 2 |
|  | AGB production change (ton/ha) | 48 | 10 | 14 | 3 |
|  | AGB productivity change (ton/ha/year) | 6.85 | 3.3 |  |  |
| **CFs** | Total AGB change (ton) | 116,788 | 29,085 | 10 | 2 |
|  | AGB production change (ton/ha) | 54 | 13 | 15 | 3 |
|  | AGB productivity change (ton/ha/year) | 7.7 | 4 |  |  |
| **Non-CFs** | Total AGB change (ton) | 149,496 | 28,132 | 10 | 2 |
|  | AGB production change (ton/ha) | 44 | 8 | 14 | 2 |
|  | AGB productivity change (ton/ha/year) | 6.2 | 2.6 |  |  |

**Appendix S5: Validation of CPA based on manually delineated tree crowns**

**
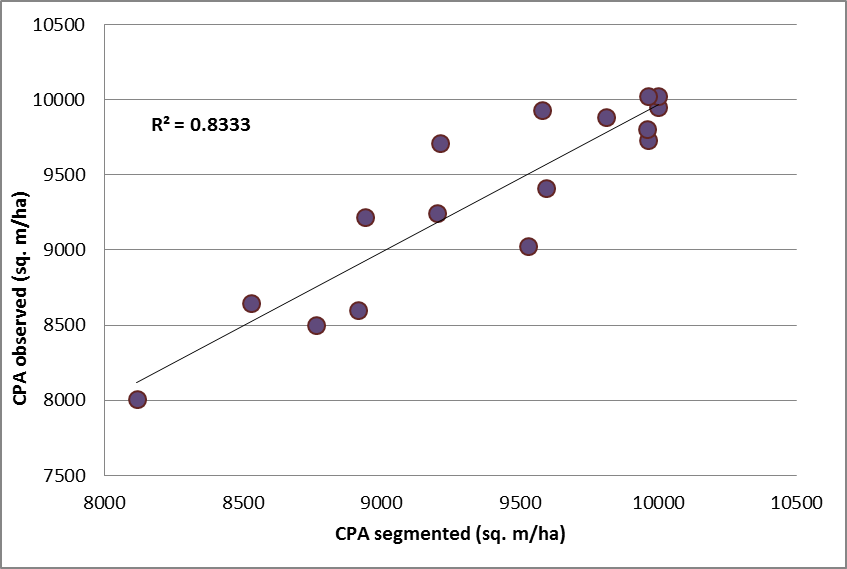
**

**Appendix S6: Crown Projection Area (CPA) maps over watershed**


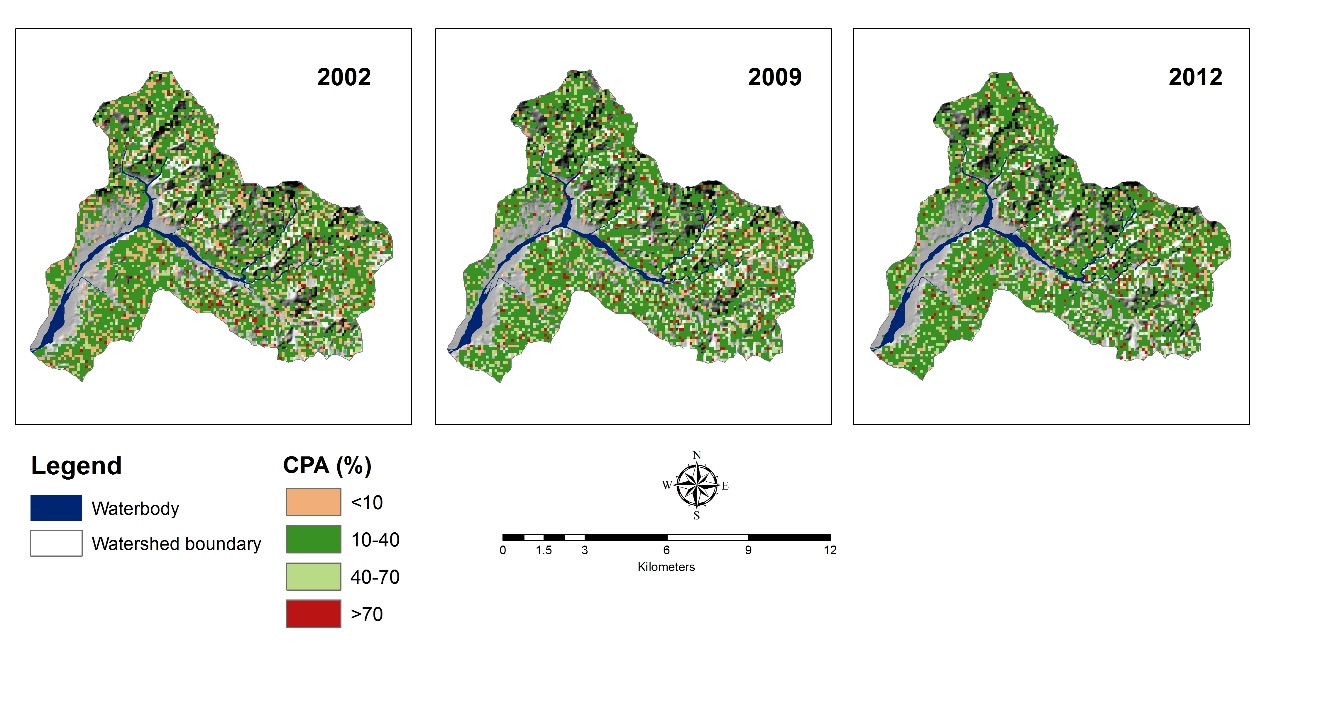


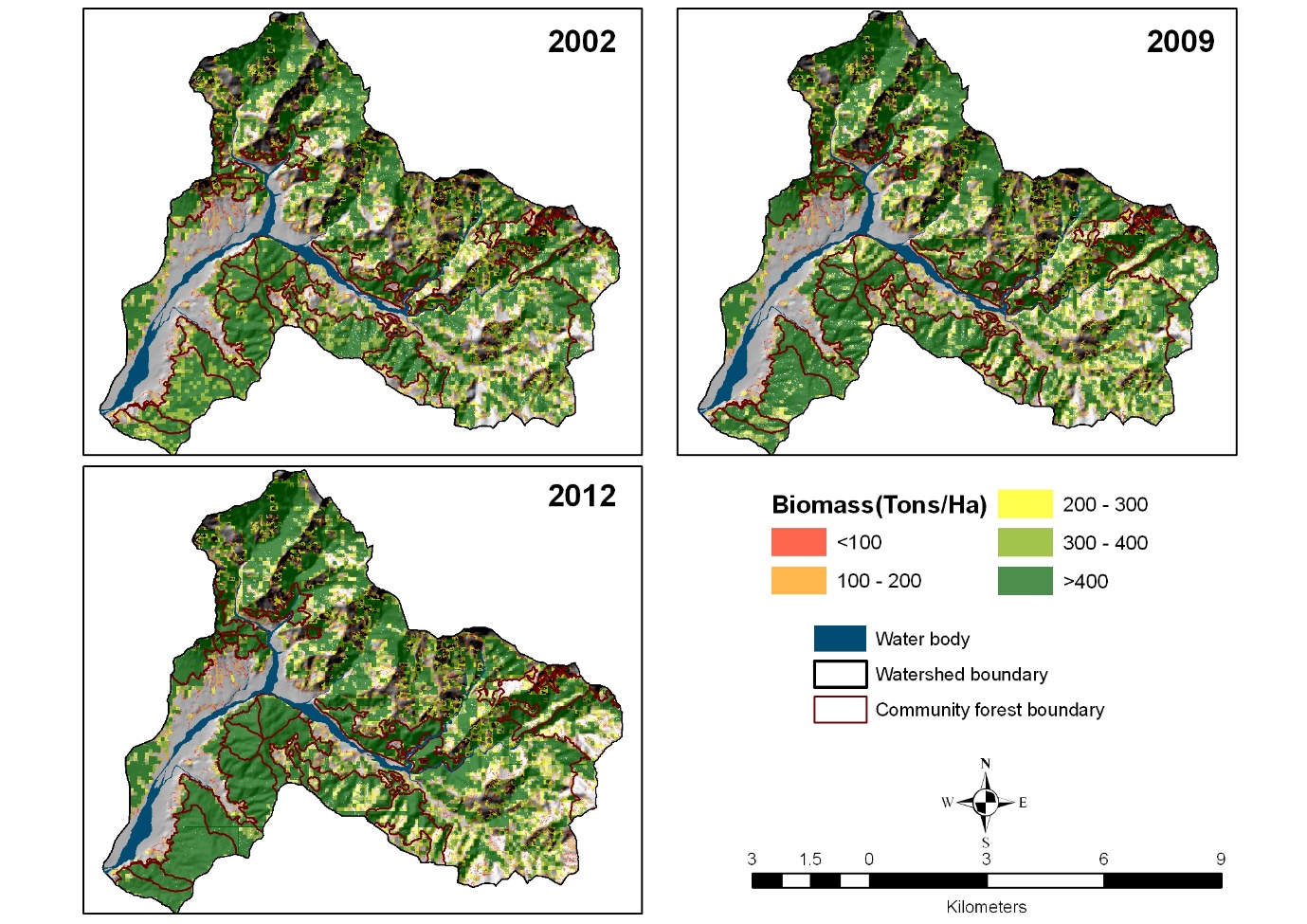
**Appendix S7: AGB maps 2002, 2009 and 2012**

**Appendix S8: Template for reading the virtual plots by the CFUGs to assess the tree canopy area in relation to basal area estimation (Grid size One ha Basal area to be multiples by 10)**

**Appendix S9: Upscaling community level monitoring to national monitoring systems using remote sensing models**

**
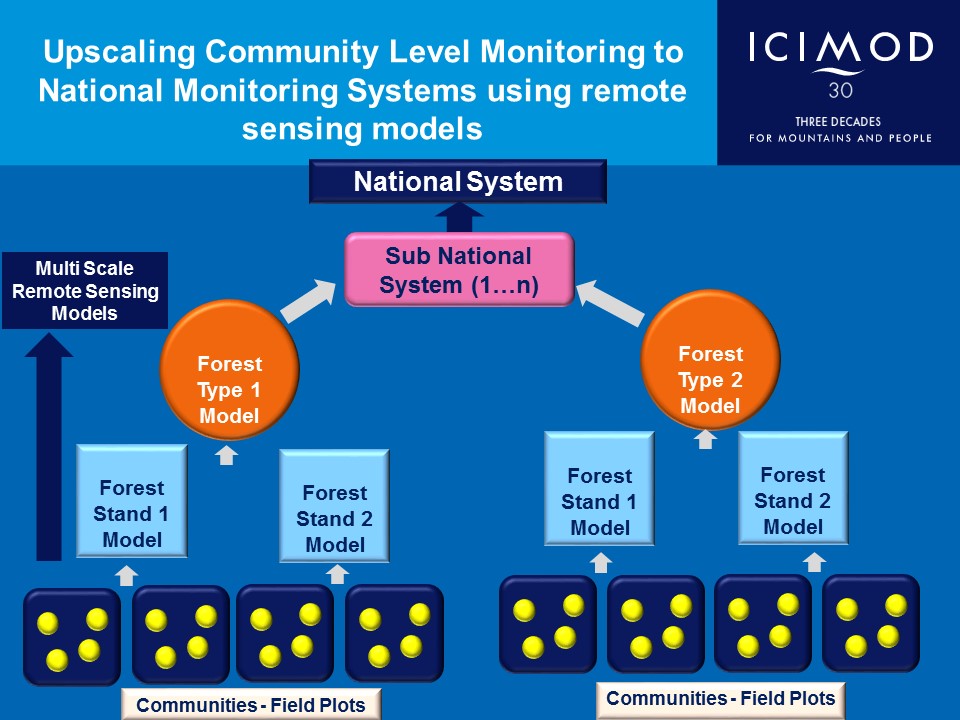
**

**Appendix S10: A comparison of forest measurement costs - Community based forest inventories in different countries**

| **Country** | **Total study area (ha)** | **Total number of plots** | **Inventory total cost (US $)** | **Cost (US $/ha)** | **Cost (US $/plot)** |
| --- | --- | --- | --- | --- | --- |
| Nepal (Our study) | 2385 | 140 | 8000 | 3.4 | 57 |
| Indonesia | 400 | 64 | 2841 | 7.1 | 44 |
| China | 761 | 60 | 2622 | 3.4 | 44 |
| Laos | 162 | 62 | 3681 | 22.7 | 59 |
| Vietnam | 314 | 103 | 14050 | 44.7 | 136 |
| India | 58 |  | 1258 | 21.7 | - |
| Tanzania | 1020 |  | 12415 | 12.2 | - |
